# Factors preventing kneeling in a group of pre-educated patients post total knee arthroplasty

**DOI:** 10.1007/s10195-016-0411-1

**Published:** 2016-05-27

**Authors:** Leigh White, T. Stockwell, N. Hartnell, M. Hennessy, J. Mullan

**Affiliations:** Faculty of Science, Medicine and Health, School of Medicine, University of Wollongong, Wollongong, NSW 2522 Australia

**Keywords:** Total knee arthroplasty, Kneeling, Patient education

## Abstract

**Background:**

Difficulties in kneeling, one of the poorest scoring functional outcomes post total knee arthroplasty (TKA),have been attributed to a lack of patient education. This is the first study to investigate specific factors affecting a patient’s perceived ability to kneel post TKA, following exposure to a preoperative kneeling education session.

**Materials and methods:**

A cross-sectional study was conducted following TKA with patients who had been educated about kneeling prior to the operation. Patients completed kneeling questionnaires at 6 (*n* = 115) and 12 (*n* = 82) months post TKA. In addition to the 12-month kneeling questionnaire, patients also completed the Oxford knee score (OKS) survey.

**Results:**

Seventy-two percent of patients perceived they could kneel at 12 months post TKA. Overall, pain and discomfort were the most common factors deterring patients from kneeling. Perceived kneeling ability was the poorest scored outcome on the OKS with patients reporting mild to moderate difficulty with this task. Kneeling scores were strongly correlated with overall knee function scores (*R* = 0.70), strongly correlated with pain scores (*R* = 0.45) and weakly correlated with knee stability scores (*R* = 0.29). When asked about other factors preventing kneeling other than pain or discomfort, 75 % had reasons unrelated to the knee or TKA. The most common reason was ‘problems with the other knee’ (*n* = 19).

**Conclusions:**

Patients in this study were provided with education regarding their kneeling ability post TKA, yet still experienced limitations in perceived kneeling ability postoperatively. Contrary to previous research, our study suggests that factors other than patient education affect a patient’s perceived kneeling ability post TKA.

## Introduction

Osteoarthritis is a leading cause of pain and disability worldwide and when present in the knee often leads to total knee arthroplasty (TKA) because of poor responses to conservative medical or physical treatments [1]. Over the past 10 years, there has been an increasing national and international demand for TKA [2–5]. Several factors have contributed to this increase. These include but are not limited to an increase in age, obesity, expectations for an improved quality of life, as well as the availability of improved and more cost-effective surgical techniques in undertaking TKA [5–7].

Satisfaction among TKA patients has increased, with as many as 81–89 % of patients being satisfied with their procedure [8, 9]. The areas of greatest satisfaction include functional areas, such as improved knee stability, reduced knee pain experienced after long periods of sitting, and improved abilities to complete basic activities of daily living [8, 9]. In comparison, areas with which patients tend to be least satisfied include pain experienced resulting from the procedure, difficulty in descending stairs and an inability to kneel post TKA [8]. The inability to kneel can be especially problematic for people who engage in activities that regularly require kneeling, e.g., recreational activities such as gardening and playing lawn bowls, and work-related tasks such as carpet laying and plumbing [10–12]. In Middle Eastern and Eastern cultures, kneeling is an integral function for everyday tasks, such as praying and sitting for meals [12, 13].

Patients want to be able to kneel after TKA [14]; however, only a few studies have examined this outcome post surgery [11, 12, 15–19]. Early research suggests that a discrepancy exists between a person’s perceived ability and actual ability to kneel [11, 15, 16]. Significant factors preventing patients from kneeling include fear of harming the prosthesis and lack of education [11, 15, 16]. There is only one small study showing a significant improvement in the ability to kneel post partial knee replacement with education sessions [20]. There are no studies investigating the effect of education sessions on the ability to kneel post TKA.

Our study investigated factors which prevent kneeling at 6 and 12 months post TKA in patients who received a preoperative education session about kneeling. The aim of this study is to determine if providing patient education prior to surgery and, therefore, provide more realistic expectations about kneeling capability post surgery has any effect on a patient’s perceived kneeling ability post TKA. The change in kneeling capabilities between 6 and 12 months after TKA will also be examined. Finally, we aimed to identify factors other than pain and discomfort, which impair the ability to kneel at 12 months post TKA.

## Materials and methods

Patients attending an outpatient clinic between July 2013 and May 2014, following their elective TKA (from the same surgeon) were invited to participate. Ethical approval was granted by the University of Wollongong Human Research Ethics Committee.

The primary indication for TKA of all volunteering patients was end-stage osteoarthritis. A midline incision with a medial parapatellar approach was used for each TKA. The patellar was resurfaced with a cemented polyethylene button and a cemented hydroxyapatite-coated posterior cruciate-retaining prosthesis was used. Circumpatellar electrocautery was performed as part of the TKA in all patients. On enrolment to the study all patients received a 30-min education session by the practice nurse. This was a standardized education session outlining what to expect on the day of TKA, what was to be expected in the recovery post-TKA period and the long-term functional prognosis of TKA. All patients were advised that they may experience pain or discomfort when kneeling and that this would not harm the prosthesis. A safe kneeling technique was also demonstrated. This education was repeated in their postoperative physiotherapy sessions. All patients received daily postoperative physiotherapy until they were deemed safe for discharge.

Patients completed two surveys, one at 6 and another at 12 months post TKA. At both 6 and 12 months, a questionnaire including patient demographics and the patient’s ability to kneel was mailed to the volunteers. The 12-month questionnaire also included a validated Oxford knee score (OKS) questionnaire [10].

The questions included in the kneeling survey included (1) Are you able to kneel on your replaced knee? (2) Do you have pain with kneeling? (3) Do you have discomfort or increased pressure within the knee with kneeling? (4) Does pain stop/prevent you from kneeling? These questions were scored as ‘yes’ or ‘no’ answers and each question was analyzed using descriptive statistical analysis. Additionally, the 12-month kneeling survey asked patients to comment on any additional factors that prevent them from kneeling or make kneeling difficult? The answers to this question were grouped into subjects for analysis.

The OKS, which was included with the 12-month kneeling survey, is a validated 12-question survey that is commonly used to gauge the functional activity and pain experienced over the past month by the patient following TKA (Table 1) [10].

**Table 1:** The Oxford knee score [10]

Question number	Question
1	Describe the pain you usually have from your knee? (pain)
2	How much trouble do you have washing and drying yourself (all over) because of your knee? (function)
3	How much trouble do you have getting in/out of your car or using public transport because of your knee? (function)
4	For how long have you been able to walk before pain from your knee becomes severe? (with or without a stick) (pain)
5	After a meal (sat at a table), how painful has it been for you to stand up from a chair because of your knee? (pain)
6	Have you been limping when walking, because of your knee? (function)
7	Could you kneel down and get up again afterwards? (function)
8	Have you been troubled by pain from your knee in bed at night? (pain)
9	How much has pain from your knee interfered with your usual work (including housework)? (pain)
10	Have you felt that your knee might suddenly ‘give way’ or let you down? (function)
11	Could you do household shopping on your own? (function)
12	Could you walk down one flight of stairs? (function)

The responses given to each of the 12 questions were given a numerical value ranging from 0 (worst) to 4 (best). From each of the responses to the OKS, we calculated overall patient perceived pain and function scores (Table 1). The overall pain and function scores were then converted into standardized scores [8]. This was performed in order to allow comparisons to be made between a patient’s knee function and knee pain. This conversion was made by dividing the overall score by the highest possible score (20 for knee pain and 28 for knee function). An example of the calculation for knee pain—total knee pain score of 15 divided by the highest possible score of 20 gives a standardized score of 0.75. The standardized scores were then subgrouped in order to compare the knee pain and function of patients who could and could not kneel.

### Statistical analysis

Pearson’s correlation coefficient analysis was used to look for correlations between the kneeling question in the OKS versus knee stability, overall knee pain and overall knee function scores. The data were then subdivided into two groups, i.e., those who could and could not kneel. Comparisons were then made within each subgroup between their standardized knee pain and function scores through multivariate logistic regression analysis. A *p* value ≤0.05 was considered to be statistically significant.

## Results

A total of 130 patients at 6 months and 98 patients at 12 months post TKA were mailed questionnaires. At 6 months, 115 (88.5 %) patients (65 female, 50 male) with a mean age of 68 years responded to the survey. Questionnaire response rate at 12 months was 83.7 % with 82 patients (46 female, 36 male) with a mean age of 69 years responding to the survey.

### Kneeling survey results

At 6 months post TKA, 73 (63.5 %) patients reported that they were able to kneel. Twenty-eight (24.3 %) patients reported that they were unable to kneel due to pain, and a further 10 (8.7 %) reported pain to a deterring factor. Discomfort and pressure in the knee were reported as deterring factors in 38.3 % of patients (*n* = 28). By 12 months post TKA, there was an increase in the percentage of patients who reported they could kneel to 72 % (*n* = 59). The number of patients reporting pain as the reason for not being able to kneel decreased to 14 (17.1 %), with 15 patients (18.3 %) reporting pain as a deterring factor. The percentage of patients reporting discomfort and pressure as deterring factors at 12 months post TKA increased to 75.6 % (*n* = 62).

When patients were asked at 12 months post TKA ‘additional factors that prevent you from kneeling or make kneeling difficult?’ eight subjects were identified. Of all the reasons given, 74 % were unrelated to the knee or complications from the TKA. The most common response was problems with the other knee as reported in 19 patients (Table 2). Only one patient reported that they felt that they could not kneel due to a fear of injuring their knee (Table 2).

**Table 2:** Factors preventing patients from kneeling at 12 months

Theme	*N*
Stiffness	5
Numbness	4
Joint effusion	1
Total	10 (26 %)
Pain in the other knee	19
Avoid kneeling because the patient has no need to/habit	7
Fear of injuring knee	1
Pain in hip	1
Overweight	1
Total	29 (74 %)
Grand total	39 (100 %)

### OKS results

**Fig. 1:**
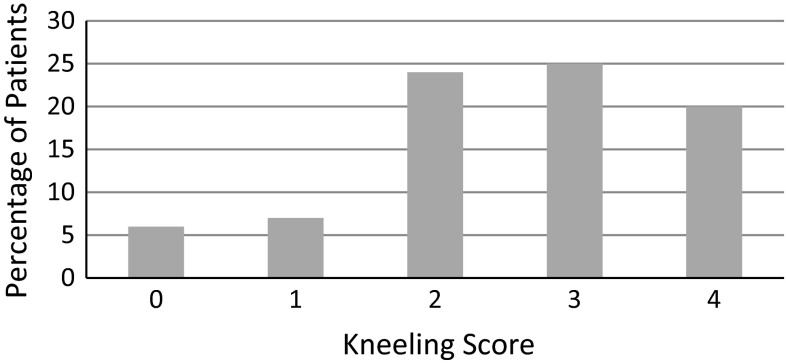
Responses to question 7 (kneeling question) in the OKS from 82 patients at 12 months post total knee arthroplasty. Score of 0 = impossible; 1 = extreme difficulty; 2 = moderate difficulty; 3 = minimal difficulty; 4 = easily

At 12 months post TKA, the average OKS was 42.5/48. The poorest reported outcome of the OKS was kneeling with patients reporting mild to moderate difficulty with this task (2.6/4; Fig. 1). Patients reported minimal to no difficulty with stairs (3.5/4) and knee stability (3.8/4), and minimal to no problems with knee pain (average score of 17.8 out of 20) and knee function (average score of 24.7 out of 28).

Pearson’s correlation coefficients (*R*) were then calculated for kneeling versus knee stability, knee function and knee pain scores. There was a very strong correlation between kneeling scores and knee function scores (*R* = 0.70), a strong correlation between kneeling and knee pain (*R* = 0.45), and a weak relationship between kneeling and knee stability (*R* = 0.29).

### Analysis of a patient’s ability to kneel and standardized knee function and knee pain scores

At 12 months post-TKA, the average standardized overall knee pain score was 0.89 and the average overall knee function score was 0.88. When comparing the scores of the patients who could and could not kneel, significant differences were detected for both standardized knee pain and knee function (*p* = 0.03 and 0.001, respectively; Table 3).

**Table 3:** Standardized knee pain and knee function scores in patients who could and could not kneel at 12 months post total knee arthroplasty

Score^a^	Overall		Able to kneel		Unable to kneel	
	No.	Standardized score (95 % CI)	No.	Standardized score (95 % CI)	No.	Standardized score (95 % CI)
Pain	82	0.89 (0.03)	57	0.91 (0.03)*	25	0.82 (0.08)*
Function	82	0.88 (0.03)	57	0.91 (0.02)^#^	25	0.80 (0.06)^#^

* Significant difference between standardized knee pain scores of patients who could and could not kneel (*p* = 0.03)

^#^Significant difference between standardized knee function scores of patients who could and could not kneel (*p* = 0.001)

^a^Pain, the summation questions 1, 4, 5, 8 and 9 of the OKS; function is the summation of questions 2, 3, 6, 7, 10, 11 and 12 of the OKS

## Discussion

Our study had a response rate of 88 % at 6 months and 83.7 % at 12 months. Groups at 6 and 12 months had comparable characteristics—115 patients (65 female, 50 male) at 6 months with a mean age of 68 and 82 patients (46 female, 36 male) at 12 months with a mean age of 69. These characteristics are comparable to previous studies on kneeling ability, which ranged in age from 66−72.2 years [11, 12, 15–20].

Patients with osteoarthritis have a poor ability to kneel preoperatively. Although this improves following TKA, patients are still expected to continue to have some difficulty kneeling [12, 21]. Only one small study (*n* = 58) has previously looked at the effect of education on kneeling post partial knee replacement [20]. This study showed limited but promising evidence that improved perceived kneeling ability was solely associated with receiving kneeling education which included a ‘one-off thirty minute physical therapy intervention and written information on kneeling’. Similar to the present study, patients were told that even though kneeling would be uncomfortable or painful it would not damage the new joint [20]. Our results showed an increase from 6−12 months post TKA in the percentage of patients who reported they could kneel from 63−72 %. In comparison, previous studies have shown kneeling ability with little or no difficultly post TKA to be between 20 and 44 % depending on the study [11, 15, 17, 18], and the percentage of patients who remain unable to kneel in these studies was 15–39 % [11, 15, 18], which is similar to our results (28–36.5 %)

The percentage of patients reporting pain as the reason for not being able to kneel decreased from 24.3 to 17.1 %. Discomfort and pressure as deterring factors at 12 months post TKA increased from 38.3 to 75.6 %. This indicates that the pain experienced by patients post TKA lessens over time and possibly becomes a residual ‘discomfort’ or ‘pressure’ in the knee. Examining discomfort post TKA would be an important area for further analysis in future studies due to the significant number of patients who are deterred from kneeling due to this experience.

There has been a growing recognition in the orthopedic field that patient-centered evaluation tools should be used to evaluate patient outcomes after TKA procedures [8]. We chose to use the OKS [10], developed over a decade ago, and which has since been demonstrated to be a suitable self-assessment tool for TKA evaluation [22, 23]. At 12 months, patients were asked to complete the questionnaire along with their kneeling survey. Consistent with the literature, the poorest reported outcome on the questionnaire was for question 7 ‘Could you kneel down and get up afterwards?’ [12, 16, 17, 24]. The majority of our patients at 12 months reported mild to moderate difficulty with this task.

We analyzed whether the ability of our patients to kneel was related to overall functional scores, pain scores and knee stability scores using the OKS [10]. There was a very strong correlation between kneeling scores and knee function scores (*R* = 0.70), a strong correlation between kneeling and knee pain (*R* = 0.45), and a weak relationship between kneeling and knee stability (*R* = 0.29). Importantly, the weak relationship between kneeling and knee stability in combination with the near perfect (3.8/4) knee stability score demonstrates that the inability of the patients to kneel was not related to an unstable knee. When comparing the scores of the patients who could and could not kneel, significant differences were detected for both standardized pain and function (*p* = 0.03 and 0.001, respectively). These results are similar to those found in a recent study in Iran for a similar-sized group of patients with osteoarthritis [18].

Previous studies evaluating kneeling ability post knee replacement have identified various factors preventing patients from being able to perform the task. For perceived inability to kneel, reasons included ‘think it may be painful’, ‘have not tried’, ‘been told not to’, ‘think it would be difficult’, ‘afraid of damaging the prosthesis’, ‘did not think they should’, ‘numbness’ and ‘stiffness’. Sixty-three percent gave reasons that could be addressed by education or rehabilitation [16]. Contrary to the common belief of these patients, there is no evidence that kneeling is harmful to the prosthesis [18, 21]. Eight subjects were identified by our patients when asked about other factors preventing them from kneeling in the survey. Importantly, 74 % of the reasons were unrelated to the knee or the surgery, including the most common response of ‘problems with the other knee’. In contrast to previous literature on this topic, only one patient had a reason normally addressed by education, which was ‘could not kneel due to a fear of injuring their knee’ [11].

There are a number of other factors such as numbness, decreased range of motion, gender [16], and choice of surgical techniques [25–27] which are mentioned in previous studies on the topic of evaluating kneeling after knee replacement, which we have not address in our current study, which would be of consideration for future studies on kneeling ability.

There are limitations to this study that need to be acknowledged. This study did not include preoperative scores of pain and functional abilities including ability to kneel. The study included a small sample size, and included patients operated on by only one surgeon at two hospital sites. The amount of information obtained through the survey was limited and may have been better addressed though an interview format. The implementation of these education sessions was not performed with a control group. Therefore, further randomized controlled trials are needed to provide a higher level evidence for this intervention. Finally not all of the patients completed the survey at 12 months.

There are several important conclusions supported by this current study. This is the first study to evaluate the effect of preoperative patient education on kneeling ability post TKA, as recommended in a number of previous studies. Even with appropriate education on kneeling ability, patients identified that pain and discomfort were significant factors preventing them from kneeling post TKA. Patients who had more pain and less overall functional ability were more unlikely to be able to kneel. This study showed an increase in the number of patients able to kneel with little or no difficulty compared to previous studies. Importantly, in contrast to previous studies, only one patient reported their reason for not kneeling as due to a fear of injuring their knee. Therefore, education sessions should be a routine part of the TKA patient journey. Consistent with previous studies, our results show that kneeling continues to be the poorest functional outcome post TKA and therefore an important area for continued research.
